# Impact of Active Recovery and Whole-Body Electromyostimulation on Blood-Flow and Blood Lactate Removal in Healthy People

**DOI:** 10.3389/fphys.2020.00310

**Published:** 2020-04-15

**Authors:** Borja Sañudo, Diego Bartolomé, Sergio Tejero, Jesús Gustavo Ponce-González, Juan Pedro Loza, Arturo Figueroa

**Affiliations:** ^1^Department of Physical Education and Sports, University of Seville, Seville, Spain; ^2^Department of Physical Education and Sports, University of Las Palmas de Gran Canarias, Las Palmas, Spain; ^3^Department of Trauma and Orthopedic Surgery, HU Virgen del Rocío, Seville, Spain; ^4^MOVE-IT Research group and Departament of Didáctica de la Educación Física, Plástica y Musical, University of Cádiz, Cádiz, Spain; ^5^Institute of Research and Innovation in Biomedical Sciences of the Province of Cádiz (INiBICA), Cádiz, Spain; ^6^Department of Kinesiology and Sport Management, Texas Tech University, Lubbock, TX, USA

**Keywords:** exercise, recovery, blood flow, electrical myostimulation, peak blood velocity

## Abstract

This study aimed to determine whether an active recovery with added whole-body electromyostimulation (WB-EMS) can increase blood flow and lead to blood lactate removal after intense exercise. Thirty-five healthy individuals (23.1 ± 4.6 years) were randomly assigned to: (a) an experimental group using active recovery together with the WB-EMS (*n* = 18) or (b) a control group using the same active recovery protocol with the suit with no-stimulation (CON, *n* = 17). Participants performed a maximal graded exercise test followed by an active recovery protocol (walking at 40% of their maximum aerobic velocity). During the recovery, participants in WB-EMS and CON received continuous stimulation at 7 Hz or no stimulation, respectively. Heart rate, blood lactate concentrations, pain/discomfort, and hemodynamic measurements were recorded before and after the test, and repeated immediately after and at min 30 and 60. The between-group analysis showed a substantially greater Peak blood velocity (−0.27 [−0.68; 0.14]) in WB-EMS compared to CON. The pain/discomfort levels were also lower in WB-EMS compared with CON (0.66 [−0.12; 1.45]). Non-significant differences in participants’ blood lactate were observed in WB-EMS compared with CON both immediately; at 30and 60 min. Our findings suggest that increased local blood flow induced by WB-EMS may have contributed to greater lactate removal from active muscles and blood lactate clearance. WB-EMS may be an effective means of increasing muscle blood flow after a maximal graded exercise test and could result in improved recovery.

## Introduction

For many years, low-frequency electrical stimulation (EMS), consisting on application of electrical stimuli via skin electrodes to induce muscle contraction, has been used for rehabilitation and recovery purposes. EMS is believed to increase blood flow leading to accelerated muscle metabolites removal (Lattier et al., 2004). EMS activates the muscle pumps of the limb resulting in significant increases in blood volume flow/velocity and skin capillary blood flow (Tucker et al., 2010). Classical studies revealed that EMS on the lower limb increased arterial flow in healthy and clinical populations (Currier et al., 1986).

This technique may have additional advantages over other recovery methods as it is easy to apply and can be used by individuals who are unable or unwilling to use other alternatives (e.g., active recovery, cryotherapy, or whole body vibration). However, despite there is no doubt that the muscle pump aids venous return due to increases in local and total blood flow (Sheriff, 2005) and reduced intracellular fluid volume (Signorile et al., 1993), the use of EMS to increase blood flow has led to contradictory results (Malone et al., 2014). It has been hypothesized that these inconsistent results could be associated with the device used or even the localization of the electric stimulation (Martin et al., 2004; Barnett, 2006). These authors suggested that the blood flow may not be effectively increased depending on stimulation characteristics. As reviewed by Babault et al. (2011), when the intensity of the stimulation is high, partial ischemia could be expected whereas a low intensity may not be enough to stimulate an increase in blood flow. Given that these devices may not cause a systemic effect, a change in the approach to improve peripheral circulation and venous return by stimulating total blood flow should be recommended (Bieuzen et al., 2012).

In this line, whole-body electromyostimulation (WB-EMS) is a new therapeutic strategy with promising results on body composition and functional capacity (Kemmler et al., 2018). This new training technology fundamentally differs from the passive and locally applied classical stimulation in that is based on electrical stimulation of large muscle groups, via fixed electrodes in the inside surface of a dedicated suit, resulting in simultaneous contraction of eight major muscles groups (Kemmler et al., 2014). However, despite the promising results in other outcomes, to date, there is still a lack of knowledge regarding the detailed acute physiological responses induced by WB-EMS on blood flow or blood lactate concentration. Therefore, in this study, we aim to show whether active recovery with a WB-EMS can alter characteristics of leg blood flow using doppler ultrasound. A further objective was to evaluate the effectiveness of WB-EMS on blood lactate removal after intense exercise. We hypothesized that active WB-EMS recovery would result in better restoration of leg blood flow and lactate clearance than an active recovery protocol alone. This may be of importance especially in sports as a variety of post-exercise recovery interventions are often employed to improve recovery from training and competition bouts (Dupuy et al., 2018).

## Materials and Methods

### Design

An overview of the experimental protocol is presented in Figure 2. This was a randomized interventional trial with all participants wearing the suit and performed a maximal graded exercise test on a treadmill followed by an active recovery exercise protocol. Active recovery has been widely recommended as a post-exercise recovery technique, reported to be effective for reducing delayed onset muscle soreness, perceived fatigue or muscle damage (Dupuy et al., 2018). Therefore, to determine if the addition of WB-EMS could bring additional benefits, during the recovery, participants randomly assigned to the experimental group were submitted to a WB-EMS continuous low-frequency stimulation (7 Hz) while control participants did not receive electrical stimulation.

**FIGURE 1:**
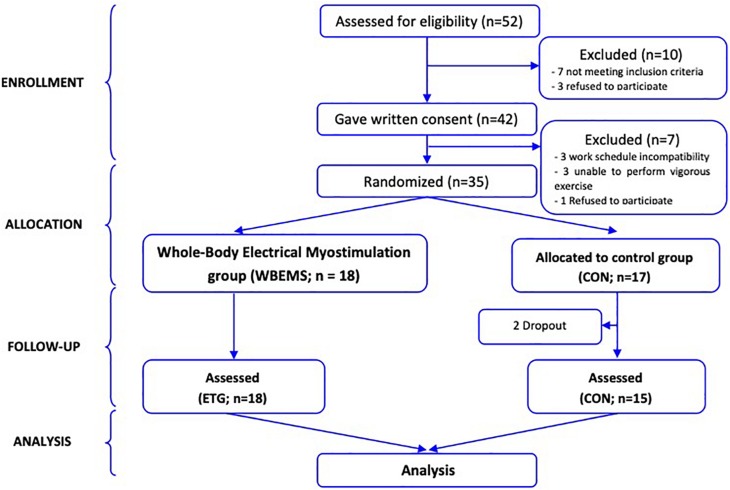
Flow of participants through the trial.

**FIGURE 2:**
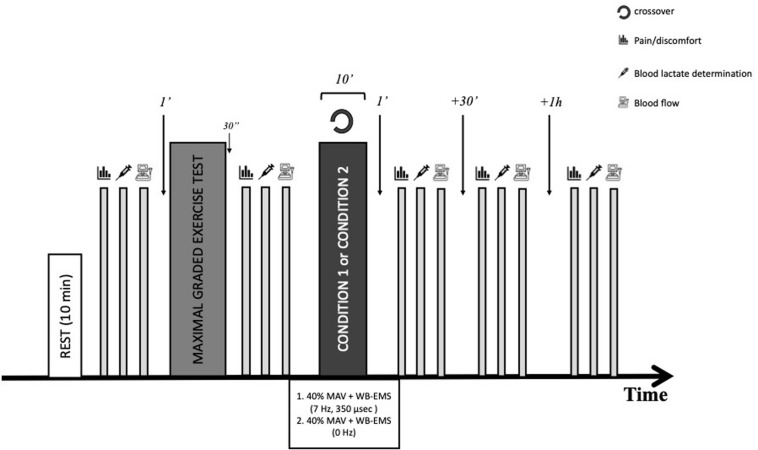
Study protocol timeline (BLa, blood lactate accumulation; MAV, maximum aerobic velocity).

Clinical details including subject age, weight, height, body composition and vascular function outcomes were collected. Laboratory personnel were blinded to group allocation. Before to start each workout, each subject wore a Polar RS800CX (Polar, Kempele, Finland), settled at beat-to-beat modality, to record heart rate during the protocol. After an equilibration rest period of 10 min, anthropometric characteristics, body composition, heart rate, blood lactate concentrations, levels of pain/discomfort, and baseline hemodynamic measurements were recorded and the participants carried out a maximal graded test on a treadmill. After that, measurements were repeated and then participants were submitted to an active recovery protocol on the treadmill at 40% of their maximum aerobic velocity (MAV). Immediately after the recovery period, hemodynamic and lactate measurements were taken in a supine position, and then, measurements were repeated at min 30 and 60 after recovery in the seated position.

### Participants

Thirty-three healthy individuals (age: 23.1 ± 4.6 years, height: 1.75 ± 0.1 m, mass: 72.0 ± 10.1 kg) volunteered to participate in the study and were randomly assigned to one of two groups: an experimental group using active recovery together with the WB-EMS with a low-frequency continuous protocol (WBEMS, *n* = 18) or a control group using the same active recovery protocol with the suit with no-stimulation (CON, *n* = 17). Individuals were excluded if they had known cardiovascular, metabolic and/or respiratory disease, or were unable to perform vigorous exercise. Only subjects with no experience using the WB-EMS were included (CONSORT diagram shown in Figure 1). Before the study, each participant was informed about the purpose and risks of the study and signed an informed consent form. The experimental protocol was conducted according to the Declaration of Helsinki statement and approved by a local ethics committee for biomedical research in Andalusia (n° 0459-N-16).

## Procedures

### Maximal Graded Exercise Test

Participants completed a graded treadmill running test until volitional exhaustion (h/p/cosmos Saturn 4.0, Traunstein Germany) with a slope set a 0°. The ramp protocol commenced at an initial speed of 1 km/h and increased at increments of 1 km/h per minute until volitional fatigue. Verbal encouragements were given throughout the test. MAV was determined as the final completed stage during the incremental protocol. Participants wore a heart rate monitor to record the maximal heart rate. The MAV was calculated using the equation proposed by Kuipers et al. (1985): MAV(*t*) = v + a^∗^(n/b) where v (in Km/h) is the velocity maintained before the last stage, a in the speed increment between two stages (Km/h), n is the number of seconds run in the last stage and b the theoretical number of seconds of this stage.

### Recovery Protocol With Whole-Body Electromyostimulation

Participants performed a recovery protocol consisted of a 10 min active recovery on the treadmill at 40% of the MAV after the maximal exercise test. While recovering, all participants had the electrodes attached on the inside surface of a dedicated suit (miha bodytec, Augsburg, Germany). This equipment enables the simultaneous activation of 10 regions (upper legs, gluteus, lower back, upper back including the latissimus dorsi, abdomen, chest, upper arms) with different intensities –mA- (mean channel tolerated intensity: 51.0, 51.0, 42.4, 52.8, 50.5, 54.9, 38.1, 25.5%). For participants allocated to WBEMS performed one bipolar protocol with continuously implicated stimulation (7 Hz) with an impulse breadth of 350 μsec. As suggested by Kemmler et al. (2016) The intensity was increased until the subject’s pain threshold and adjusted during the recovery protocol every 2 min using ratings of perceived exertion (Borg CR10-Scale). Participants in the CON group performed the same recovery protocol but without stimulation (0 Hz).

## Measurements

### Anthropometric and Body Composition Measurements

The height and weight of the subjects were measured and body mass index (BMI) was then calculated by dividing weight (kg) by height squared (m^2^). Bio-electrical impedance was used to determine body composition using Bodystat©1500 (Bodystat Ltd., Douglas, Isle of Man, United Kingdom), which is a four terminal single frequency (500 μA at 50 kHz) analyzer. Resistance and reactance were measured between the right wrist and the right ankle and total body fat, fat-free mass, and their percentages were estimated from the manufacturers’ equations (Table 1).

**TABLE 1:** Characteristics of the participants in the study (*n* = 33).

	WBEMS	CON	*p*
Age	22.22.1	24.36.6	0.184
Weight	73.618.15	70.8612.38	0.448
Height	1.760.11	1.730.11	0.341
BMI	23.911.86	23.602.55	0.687
Fat mass	16.207.50	15.866.90	0.895
HR rest	61.798.67	61.149.15	0.838
HR max	190.118.43	188.149.25	0.535
MAV	16.781.63	16.491.52	0.606
40%MAV	6.710.65	6.600.61	0.606

*MAV, maximal aerobic velocity; HR, heart rate; BMI, body mass index.*

### Baseline Ultrasound

Subjects fasted for 8 h, abstained from alcohol and caffeine for 12 h, and did not perform any exercise for 24 h before assessments. Participants were positioned in prone position on a stretcher for comfort during the scanning protocol and after a rest period of 10 min, measurements were recorded on the dominant leg using a ultrasound machine (SonoSite S-Nerve, SonoSite Iberica SL, Madrid, Spain) and a linear high-frequency ultrasound transducer (6–13 MHz, HLX38) in a quiet and environmentally controlled room. In all ultrasound procedures a preset optimized vascular protocol was used. The superficial femoral artery and superficial femoral vein were identified (about 2 cm proximal to the bifurcation of the common femoral artery) and the position of the probe was marked on the skin for consistency of repeat measurements.

All blood flow measurements were taken in triplicate and the mean was considered for later analysis. Blood flow outcomes measured were Peak blood velocity (PBV), resistive index (RI) and pulsatility index (PI). Acceleration time to peak flow (TA) was also recorded. These outcomes were automatically calculated and were then manually recorded by the investigators.

### Blood lactate and pain/discomfort levels

Blood samples were collected from the right earlobe to determine lactate concentrations. Blood lactate concentrations were measured using a lactate analyzer (Lactate Pro 2; Arkray, Kyoto, Japan) at baseline, following the maximal graded exercise test, immediately after the recovery protocol and 30 min and 1 h following the recovery. At the same time-points the level of pain/discomfort was assessed with a 100-mm visual analog scale (VAS).

### Data Analysis

Descriptive statistics were calculated at baseline for demographic variables and dependent measures with all *p* < 0.05 considered statistically significant. Group by measures interactions were determined by using a single-factor repeated measures analysis of variance. All data were first log-transformed to reduce bias and then effect size (ES, 90% confidence limit) was calculated using the pooled pre-exercise SD. The threshold values used for ES were >0.2 (small), >0.6 (moderate) and >1.2 (large) as suggested by Hopkins et al. (2009). For between-groups comparisons, the chances that the differences in recovery were better/greater, similar, or worse/smaller as well as the quantitative chances (beneficial/better or detrimental/poorer effect) were calculated as follows: <1%, almost certainly not; 1–5%, very unlikely; 5–25%, unlikely; 25–75%, possibly; 75–95%, likely; 95–99%, very likely and >99%, almost certainly (Hopkins et al., 2009). If the chances of having beneficial/better and detrimental/poorer performances were both >5%, the true difference was considered unclear.

## Results

Characteristics of study participants are reported in Table 1. Two participants dropped out in CON (did not attend the final assessment session); therefore, data from 33 participants were analyzed. There were no significant differences in all measurements between WBEMS and CON groups at baseline. Their mean (standard deviation) age was 23.2 ± 4.3 years and BMI 23.8 ± 2.2 kg/m^2^. The mean MAV was 16.6 ± 1.6 km/h.

Table 2 shows the between-group differences after the maximal graded exercise test and the subsequent recovery on vascular function and blood lactate removal. The analyses showed a substantially greater PBV (−0.27 [−0.68; 0.14], 3/36/61%) in WBEMS compared to CON. After 60 min of the recovery process, RI decreased substantially more in WBEMS than in CON *likely* due to the intervention. Further, the pain/discomfort levels at this point were also lower in WBEMS compared with CON (0.66 [−0.12; 1.45], 84/12/4%). Non-significant differences in participants’ blood lactate were observed in WBEMS compared with CON both immediately, at 30 and 60 min after the maximal graded exercise test. Non-significant differences were found between-groups in blood lactate removal during the time-course of the recovery. WBEMS participants increased the blood lactate levels (from 0.92 ± 0.24 to 8.19 ± 2.45 mmol/l) by 15% more than CON participants (from 1.00 ± 0.21 to 7.19 ± 2.58 mmol/l) and these levels remained high during the recovery.

**TABLE 2:** Between-group analysis during the WB-EMS and active recovery intervention on vascular function and lactate removal.

Outcome	Recovery	Standardized differences (CL90%)	% (CL90%)	Chances	Outcome
PBV (cm/s)	Immediately	−9.1(−21.6;5.3)	−0.27(−0.68;0.14)	3/36/61%	Possibly
	30 min	−2.4(−20.6;20.0)	−0.07(−0.64;0.51)	22/44/35%	Unclear
	60 min	−1.7(−24.1;27.3)	−0.05(−0.75;0.66)	28/37/36%	Unclear
RI	Immediately	5.4(−31.4;61.9)	0.06(−0.46;0.59)	33/47/20%	Unclear
	30 min	8.9(−9.0;30.3)	0.24(−0.27;0.75)	56/37/7%	Unclear
	60 min	−17.4(−36.9;8.1)	−0.23(−0.55;0.09)	2/42/56%	Possibly
PI	Immediately	−6.1(−57.9;109.2)	−0.06(−0.85;0.73)	28/33/38%	Unclear
	30 min	2.0(−39.4;71.6)	0.02(−0.49;0.53)	28/49/23%	Unclear
	60 min	27.9(−40.1;173.3)	0.22(−0.45;0.89)	52/33/15%	Unclear
TA (ms)	Immediately	6.5(−43.9;102.2)	0.07(−0.67;0.82)	39/34/27%	Unclear
	30 min	25.6(−33.7;137.8)	0.26(−0.48;1.01)	56/29/15%	Unclear
	60 min	18.5(−37.6;124.9)	0.19(−0.54;0.93)	49/32/18%	Unclear
Lactate (mmol/l)	Immediately	−24.7(−39.8;−5.8)	−0.86(−1.53;−0.18)	1/5/95%	Very likely
	30 min	−22.7(−35.1;−7.9)	−0.78(−1.31;−0.25)	0/3/96%	Very likely
	60 min	−29.8(−42.3;−14.6)	−1.03(−1.60;−0.46)	0/1/99%	Very likely
Pain/discomfort (0–10)	Immediately	−15.9(−58.3;69.6)	−0.19(−0.96;0.58)	20/31/49%	Unclear
	30 min	52.5(−32.8;245.6)	0.46(−0.43;1.36)	69/20/11%	Unclear
	60 min	85.3(−10.6;283.7)	0.66(−0.12;1.45)	84/12/4%	Likely

*PBV, Peak blood velocity; RI, resistive index; PI, pulsatility index; TA, Acceleration time to peak flow.*

## Discussion

To our knowledge, this study is the first that has proposed to determine the impact of WB-EMS as an active recovery strategy after maximal exercise to improve femoral artery blood flow and blood lactate removal in healthy participants. This study demonstrated that WB-EMS *possibly* increased PBV when compared to CON. These changes were accompanied by a similar change (ES: −0.86) in blood lactate immediately after a maximal graded exercise test and also after 30 and 60 min of recovery (ES: −0.78 and −1.03, respectively).

These results may be particularly important for sports performing successive rounds (e.g., rowing) or during half-time in team sports. After high-intensity exercise a complete recovery is needed for a return to homeostasis what normally occurs within an hour or shortly thereafter (Malone et al., 2014). To date the impact of the various recovery protocols on blood flow and lactate removal following maximal exercise have been investigated in previous research; however, while some preliminary results suggested that local neuromuscular electrical stimulation (NMES) improves recovery from intensive exercise in professional team sports players, other have showed contradictory results (Erten et al., 2016). Recently, Malone et al. (2014) assessed the acute effects of NMES on post-exercise recovery and was considered a strategy able to increase blood flow; however, it was reported to be less effective than an active recovery. In fact, most of the studies based on electrostimulation as a recovery strategy have reported increased blood flow and metabolite washout from muscles (Lattier et al., 2004) but mainly in the supine position. The studies based on active recovery with this technology are less common; moreover, the studies that have investigated the effects of different electrical stimulation protocols on recovery were normally used at intensities insufficient to induce significant muscle contractions. Only a couple of studies (Martin et al., 2004) investigated the effects of NMES on recovery from exercise using continuous protocols enough to achieve visible muscle contractions. However, as stated above, the efficacy of NMES was not greater than traditional recovery intervention modalities.

The results on blood cell velocity reported in the current study are consistent with those previously showed after NMES (Williams and Flynn, 2014), suggesting that these stimulus may be an effective measure for enhancing blood flow rates. The underlying physiological mechanisms that may account for the efficacy of WB-EMS include muscle pump activation to increase venous blood flow (Kaplan et al., 2002). Although the duration of WB-EMS protocols has been studied in previous studies (Griffin et al., 2010), there is no consensus regarding the intensity. In the current study a continuous bipolar stimulation (7 Hz, impulse width: 350 μsec) was imposed and the intensity was increased until the subject’s pain threshold (mean channel tolerated intensity from 25.54 ± 7.25 to 54.92 ± 10.90%). Previous protocols including NMES suggested that these low-frequencies (<10 Hz) are associated with high-intensity stimulations (Babault et al., 2011); however, only one previous study analyzed the effects of WB-EMS on blood velocity (Menendez et al., 2015). In this study the current intensity was also increased until the subject’s pain threshold (mean tolerated intensity: 47.4 ± 11.2 mA) and although the protocols are not comparable (a rectangular, biphasic and symmetric wave with 8 Hz; 400 μs), authors suggested that this type of technology was able to enhance the mean blood velocity and PBV of the popliteal artery. However, only when WB-EMS was combined with whole body vibration, PBV was maintained above baseline values during the first minute of the recovery period.

Considering that the number of muscles stimulated by the WB-EMS are greater than those stimulated by NMES, one may speculate that the effect (e.g., muscle pump) would be increased. However, besides the muscle discomfort associated to the increment of intensity, WB-EMS can over-activate muscle fibers and induce damage (as reviewed by Nosaka et al. (2011). In fact, cases of rhabdomyolysis have been reported, even in highly trained athletes, due to the use of this type of technology (Nosaka et al., 2011; Kastner et al., 2015). By contrast, an interesting aspect to consider in our study was that the levels of pain/discomfort were lower in the WBEMS group than in the CON group likely showing lower fatigue perception 60 min after the test to exhaustion, with an 84% chances for a better recovery when wearing the suit with WB-EMS on than off. Although positive, these results could indicate that the stimulation may not have been intense enough.

In the same line, higher intensities of NMES stimulation would result in a greater muscle activation, which may lead to a faster metabolite removal as reviewed by Malone et al. (2014). In the current study it seems that active recovery together with WB-EMS contributed to increase the metabolism and blood flow of working muscles. However, while a metabolic removal (e.g., lactate) could be expected (Toubekis et al., 2008; Erten et al., 2016), interestingly, in the present study, lactate remained above the concentrations recorded in CON even 60 min after the end of the intense exercise, although these changes were not statistically significant. These results are in agreement with a recent study (de la Camara Serrano et al., 2018) which also did not observe significant differences between WB-EMS, active or passive recovery in blood lactate after the intense exercise; however, showed high values after WB-EMS at 20 min of recovery. One possible explanation to these unexpected results can be attributed to the electrical stimulation intensity, as suggested by Babault et al. (2011). These authors proposed two different types of effects that could be expected: (a) a low intensity that would not be sufficient to induce changes in the blood flow or, (b) an excessive intensity that might lead to partial muscle ischemia. However, there was an increase in PBV inmediately after the test that it could hardly be sustained with this argument. In view of previous results where “strong but comfortable” low frequency stimulation was used (Lattier et al., 2004), metabolites washout such as lactate can be expected (Neric et al., 2009). But generally, these washout effects were attributed to the electro-induced muscle blood flow increase (Vanderthommen and Duchateau, 2007). Probably, the greater number of muscles stimulated evoked an acute effect on blood flow, but also on systemic fatigue with the consequent maintenance of high lactate values. In this line, Borne et al. (2017) suggested that NMES would contribute to lactate removal from the exercising muscles. In contrast, authors indicated that applying a systemic treatment might decrease the effectiveness to remove lactate from the active muscle during exercise. It is possible that the increased blood flow during WB-EMS may have promoted greater lactate removal from active muscles and clearance from the blood, resulting in similar levels during the recovery period. In any case, while WB-EMS would improve the peripheral artery blood flow and the venous return, results on peripheral fatigue from metabolite accumulation are inconclusive.

There are a number of limitations to be considered when interpreting the results. A major limitation of this study is that the fatiguing exercise produced different muscle responses (e.g., soreness levels) due to subjects’ characteristics. Further, the absence of positive effect in some outcomes could partly be attributed to methodological concerns such as the arbitrary choice of stimulation intensity and chosen duty cycle. While in previous studies investigating WB-EMS protocols no information about the level of impulse intensity (mA) was given (Pano-Rodriguez et al., 2019), it should be recognized that the level of intensity needed to induce muscle contractions varied significantly in each participant. Moreover, the response to these types of stimulations used to show a high degree of inter-individual variability which lead to time-courses of recovery and a high degree of variability between subjects in the levels of fatigue. Further, gender was not considered as a factor affecting the efficacy of WB-EMS despite that previous studies associated the male gender with higher peak systolic velocities and higher ejected total volume per minute in the popliteal veins (Griffin et al., 2010). Despite these limitations, it can be concluded that WB-EMS may be an effective means of increasing muscle blood flow after a maximal graded exercise test in healthy individuals and could result in improved perception of recovery (reductions in muscle pain/discomfort). The increased blood lactate after WB-EMS, although non-significantly different from control, may indicate greater removal from active muscles.

## Perspectives

In view of our results, this study could have a number of important practical implications for physical performance as WB-EMS can be easily included to improve recovery following intensive exercise and can do it to a greater extent than a traditional active recovery. Therefore, WB-EMS could be used in situations in which traditional strategies are not possible, especially in sporting events using short recovery periods, successive rounds or during half-time in team sports that could improve the blow flow that it would increase lactate removal after high-intensity exercise.

## Data Availability Statement

The datasets generated for this study are available on request to the corresponding author.

## Ethics Statement

The studies involving human participants were reviewed and approved by Ethics committee for biomedical research in Andalusia (n° 0459-N-16). The patients/participants provided their written informed consent to participate in this study.

## Author Contributions

BS, DB, and JP wrote the manuscript. BS and DB conceived and designed the study. JP-G and JL recruited the subjects. ST and AF analyzed the data. All the authors reviewed the manuscript.

## Conflict of Interest

The authors declare that the research was conducted in the absence of any commercial or financial relationships that could be construed as a potential conflict of interest.
